# Contemporary perspectives of core stability training for dynamic athletic performance: a survey of athletes, coaches, sports science and sports medicine practitioners

**DOI:** 10.1186/s40798-018-0150-3

**Published:** 2018-07-16

**Authors:** David R. Clark, Michael I. Lambert, Angus M. Hunter

**Affiliations:** 1 School of Sport and Exercise Sciences, Faculty of Science, Liverpool John Moore’s University, 102, 2 Moorfields, Liverpool, L2 2BS UK; 2 Division of Exercise Science and Sports Medicine, Department of Human Biology, University of Cape Town, Cape Town, South Africa; 3 Physiology, Exercise and Nutrition Research Group, Faculty of Health Sciences and Sport, University of Stirling, Stirling, UK

**Keywords:** Core, Stability, Dynamic, Trunk, Athletic, Performance, Loaded, Functional, Compound, Exercise

## Abstract

**Background:**

Core stability training has grown in popularity over 25 years, initially for back pain prevention or therapy. Subsequently, it developed as a mode of exercise training for health, fitness and sport. The scientific basis for traditional core stability exercise has recently been questioned and challenged, especially in relation to dynamic athletic performance. Reviews have called for clarity on what constitutes anatomy and function of the core, especially in healthy and uninjured people. Clinical research suggests that traditional core stability training is inappropriate for development of fitness for heath and sports performance. However, commonly used methods of measuring core stability in research do not reflect functional nature of core stability in uninjured, healthy and athletic populations. Recent reviews have proposed a more dynamic, whole body approach to training core stabilization, and research has begun to measure and report efficacy of these modes training. The purpose of this study was to assess extent to which these developments have informed people currently working and participating in sport.

**Methods:**

An online survey questionnaire was developed around common themes on core stability training as defined in the current scientific literature and circulated to a sample population of people working and participating in sport. Survey results were assessed against key elements of the current scientific debate.

**Results:**

Perceptions on anatomy and function of the core were gathered from a representative cohort of athletes, coaches, sports science and sports medicine practitioners (*n* = 241), along with their views on effectiveness of various current and traditional exercise training modes. Most popular method of testing and measuring core function was subjective assessment through observation (43%), while a quarter (22%) believed there was no effective method of measurement. Perceptions of people in sport reflect the scientific debate, and practitioners have adopted a more functional approach to core stability training. There was strong support for loaded, compound exercises performed upright, compared to moderate support for traditional core stability exercises. Half of the participants (50%) in the survey, however, still support a traditional isolation core stability training.

**Conclusion:**

Perceptions in applied practice on core stability training for dynamic athletic performance are aligned to a large extent to the scientific literature.

**Electronic supplementary material:**

The online version of this article (10.1186/s40798-018-0150-3) contains supplementary material, which is available to authorized users.

## Key points

- Core stability training for healthy and athletic populations has recently been questioned and challenged in scientific literature. The narrow definition of both the anatomy, spinal region between pelvis and diaphragm, and the method of training the core through the isolation of muscles in this region does not relate to full body core function that characterises dynamic athletic performance.
- The survey reveals that this is reflected in opinions of people working and participating in sport. Half of the participants identified the area between and including the pelvic and shoulder girdles as the core. Majority supported functional loaded exercises such farmer’s walk (87%) and barbell squats (84%) as effective exercises for the development of core stability.
- Despite the support for a more functional approach, selected traditional core stability training methods do retain a certain amount of support; isometric plank exercise (56%) and unstable stability ball exercises (41%). Many respondents (42%) felt that core function should be measured subjectively through observation of sporting and or exercise performance.
- Trunk is the preferred name of the anatomical region for almost half (45%) the participants while 35% supported the term core.

## Background

The absence of a universally accepted definition of core stability (CS) is well noted in the scientific literature [1–8]. A number of these publications have proposed a definition, focussing either on function, anatomical constituents of the core or both. Several reviews have questioned and challenged core stability training (CST) for prevention and treatment of back pain [9–11] and for improvement of function and performance in healthy and athletic populations [1, 5–7, 12–14]. There is a view [1, 7] that CST in its current form evolved from clinical research [15] in the 1990s. The application of a clinical exercise approach in healthy and athletic populations has been criticised, primarily on the basis that teaching an isolated muscle pattern in uninjured athletes is unfounded [6, 10, 16]. Despite this, CST as an intervention spread to all exercise disciplines across clinical, fitness and sports performance settings with significant commercial interest and support [14].

Most review articles on this topic recognised that the application of traditional CST in healthy and athletic groups lack scientific justification [3, 7, 14, 17]. This resulted in a body of research investigating CST in healthy populations [18–22] along with aforementioned review articles [1, 6, 7, 12–14]. Reviewers have noted that research cannot progress this topic effectively until there is a standardised agreement on the anatomical structure and function of the core [1, 6, 7]. A further limitation reported by most reviewers is the absence of a valid and reliable test of core function [1, 12]. As a result most research on the topic is methodologically limited [12, 13] and therefore ineffective in confirming or challenging the concept and practice of CST for health and performance. A case has been made in the literature for a more functional definition of anatomy of the core, applicable to healthy and athletic populations [1, 8]. Similarly, it is proposed that the description of core function is revised to encompass normal healthy and athletic human movement [8].

Several comprehensive reviews over the last decade have examined the research on the effectiveness of various CST methods for athletic performance [1, 6, 7, 12–14]. Reviews covered the variations in CST including instability training, trunk rotation exercises, functional training and exercise intensity. Martuscello et al. proposed a five core exercise classification system based on their review of the research [6]. The categories were traditional core exercise (sit-ups), core stability exercises (isometric plank), ball or device exercises (stability ball), free weight exercise (squat and deadlift) and noncore free weight exercise (upper body). In a recent study conducted in an applied performance sport setting, Spencer et al. proposed a comprehensive spinal exercise classification [2]. The classification incorporated static and dynamic exercises that were either functional or non-functional according to spinal displacement across four physical outcomes: mobility, motor control, work capacity and strength. Both studies [2, 6] clarify the range and nature of core stability exercises used in the literature and practice; however, there is concern that many core stability intervention studies are diluted by other exercises and activities preventing a clear assessment of impact of CST [7, 12, 13]. Furthermore, in athletic populations, a reductionist approach or selective activation to improve integrated function is unsubstantiated [1, 2, 7, 12].

The proposed protection against injury and improved athletic performance from CST has been the subject of many research studies and review papers. Silfies et al. concluded that following a review of 11 studies, there was limited evidence to support the use of CST to prevent upper extremity injury and improve athletic performance [3]. The authors questioned whether performance in core stability tests reflected physical or athletic capability and level of conditioning, rather than solely core stabilization. Tests included the isometric front and side bridge, single-leg raise [10], star excursion test [11] and closed kinetic chain upper extremity stability test [12]. A systematic review conducted by Prieske et al. [12] concluded that CST compared with no training or regular sports-specific training does improve trunk muscle strength measured predominantly by isometric plank. However, increases in trunk muscle strength only had a small effect on physical fitness and athletic performance measures in trained individuals. CST compared to alternative physical training methods in trained individuals had little impact on trunk muscle strength, physical fitness and athletic performance measures. Both studies strongly suggest that high levels of general fitness are associated with better performance in CS tests and therefore a lower risk of injury and better athletic performance test scores [3, 12].

Separating the core into smaller local and larger global muscles has little bearing on core stability for dynamic movement in healthy people. In Lederman’s [10] words, this is an anatomical classification with no functional relevance. The role the core plays in stabilising the body is dynamic and responsive to many postural challenges that occur in normal movement and complex, reactive environment of sport [14]. The concepts of core strength and core stability have been reviewed the literature [1, 5, 23]. Whether these are separate attributes [5] or whether core strength is required for core stability [23] remain unresolved questions [1]. In this context, core stability is an integrated, functional motor task [7, 24] and training should reflect this according to movement patterns [14, 24], forces [7, 24] and torque and velocity [8, 24].

A limitation identified by Prieske et al. [12] was the lack of validity of tests used in most of the research. Trunk muscle strength in most studies was measured by timed isometric test (prone bridge) which, firstly, does not reflect force and velocity of movement of dynamic athletic activity [12]*.* Secondly, CST programmes in many of the studies incorporated prone plank or similar isometric exercises in the exercise intervention, which rendered timed isometric prone plank an inappropriate test of trunk muscle strength in these cases. Most reviews conclude there is not a valid method of measuring the effect of CST on trunk muscle strength within the context of improving dynamic athletic performance [1, 13, 14, 17, 25, 26]. As a result, many researchers have resorted to using conventional performance tests such as countermovement jump and sprint tests [12, 13, 27].

The first three levels of Martuscello’s [6] core exercise classification system appear to contravene the established overload training principle [28] when applied to an athletic population. Traditional low load core exercises, minimal range or isometric core stability exercises and ball/device exercises are all characterised by low force, low velocity and restricted range of movement. Hence, these do not represent training overload in preparation for activities that characterise most sports and athletic events. Researchers have begun to investigate trunk muscle activation in a number of dynamic, loaded free weight exercises to determine their suitability for the development of dynamic trunk strength and stability [29–37]. Surface electromyography methodology shows there is good evidence that loaded exercises performed in a standing position are an effective method of overloading the trunk stabilization system in a dynamic manner. While several reviewers recognise this development [6, 7, 14], it is best summarised by Wirth et al. (2016), ‘… we recommend the use of classical strength-training exercises as these provide the necessary stimuli to induce the desired adaptations.’

The flawed foundations of CST for dynamic athletic performance have been exposed in the scientific literature. Research is underway to better understand the most effective training methods for the development of trunk stability. The aim of this survey is to assess the current perspectives of CST in the applied sports setting to determine how well scientific literature informs these opinions. Our hypothesis is that opinions of those who work and participate in sport will reflect scientific debate on key core stability training topics.

## Methods

The online survey questionnaire (Additional file 1) was developed around common themes on core stability as defined in the current scientific literature. The online survey was created and distributed using Bristol Online Survey (BOS) tool (Tower Hill, Bristol, UK). The questionnaire comprised four sections: anatomy of the core, function of the core, methods of measuring core function and methods of training the core. The survey concluded with general questions about the application of core strength training for dynamic athletic performance.

The survey question on the anatomy of the core is based on definitions in the literature. We used the definition of local and global stabilization of intersegmental spine proposed by Bergmark (1989) [38]; the passive spinal column, active spinal muscles and neural control unit as described by Panjabi [39]; axial skeleton between pelvic and shoulder girdle including rib cage, spinal column and associated muscle and nerves proposed by Behm et al. [8]; and lumbo-pelvic hip complex according to Faries and Greenwood [23]. Categories of exercises and selection criteria for CST used in the survey question were drawn from published studies that investigated muscle activation using these manipulations. The question around core strength and core stability were based on reviews of this topic [1, 7].

A pilot survey was conducted using the postgraduate sports studies group (*n* = 20) at the University of Stirling. The questionnaire was modified according to feedback from the pilot survey. Approval for the study was granted by the local research ethics committee in accordance with the Helsinki Declaration (2013) [40].

### Participants

The survey was circulated using two methods: shared with the principal authors’ 700 LinkedIn connections and sent by email to 220 qualifying contacts. All recipients were asked to share the survey with all their contacts that met the criteria of working or participating in sport.

### Statistical analysis

**Fig. 1:**
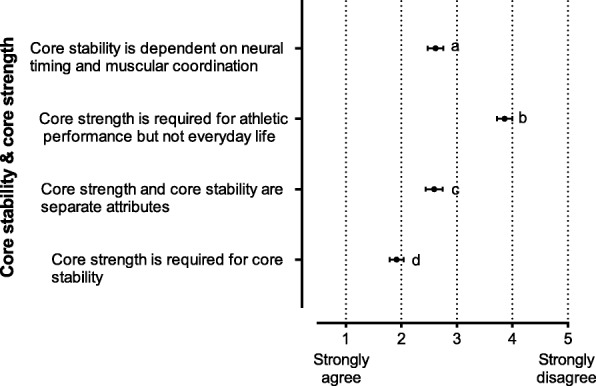
Reported support for a series of statements relating to core stability and core strength. Data are reported as mean level of agreement with 95% CI. 1 = strongly agree, 5 = strongly disagree. Significant differences *p* < 0.001: a vs b, a vs d, b vs d and c vs d. CI: confidence interval

**Fig. 2:**
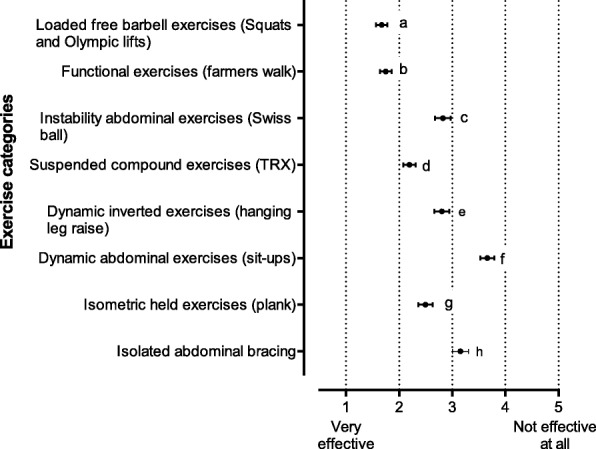
Responses to a series of questions on the effectiveness of selected categories of exercise in developing core stability for dynamic athletic performance. Data are reported as mean level of effectiveness with 95% CI. 1 = very effective, 5 = not effective at all. Significant differences *p* < 0.001: a vs c, d, e, f, g and h; b vs c, d, e, f, g and h; c vs d and f; d vs e, f and h; e vs f; f vs g and h; g vs h. CI: confidence interval

**Fig. 3:**
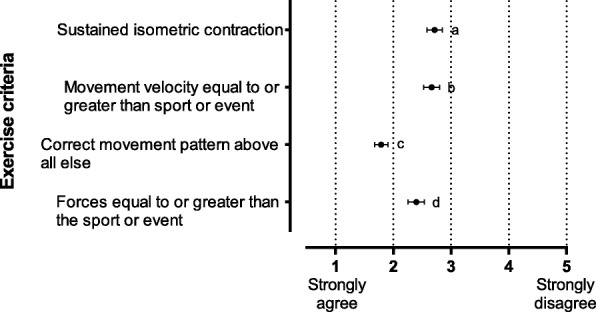
Responses to which criteria should inform exercise selection for the development of core stability for dynamic athletic performance. Data are reported as mean level of agreement with 95% CI. 1 = strongly agree, 5 = strongly disagree. Significant differences *p* < 0.05: a vs c, a vs d, b vs c, b vs d and c vs d. CI: confidence interval

**Fig. 4:**
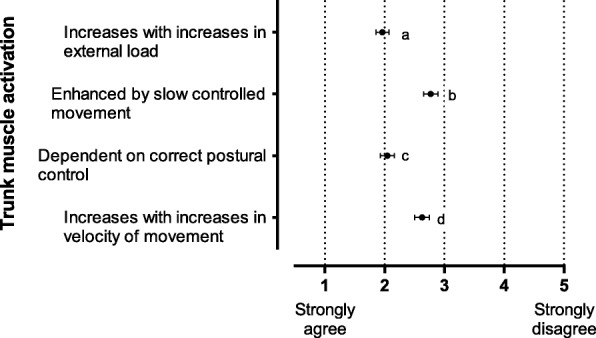
Responses to a series of statements relating to ground-based loaded free barbell exercises and trunk muscle activation. Data are reported as mean level of agreement with 95% CI. 1 = strongly agree, 5 = strongly disagree. Significant differences *p* < 0.001: a vs b, a vs d, b vs c, c vs d. CI: confidence interval

The data analysis was descriptive and frequency was presented in the tables as number and percentage (*n* (%)). Data presented in Figs. 1, 2, 3 and 4 were analysed using Kruskal-Wallis test to assess support for each statement on 5-point Likert scale. Data presented as mean and 95% CI. Five-point scale is as follows: 1 = strongly agree or very effective and 5 = strongly disagree or not effective at all. Significant differences were further analysed using Dunn’s multiple comparison post hoc test. Priori alpha level of significance was set at *p* < 0.05.

## Results

### Participants

The online survey was completed by 241 respondents from a range of disciplines involved in sport (Table 1). The highest return by employment group was received from strength and conditioning coaches (S&CC; 47%) followed by athletes and players (A&P; 17%) and sport medicine practitioners and physiotherapists (SM&P; 17%). A quarter of the cohort were involved in sport at university or school level (27%). A similar number (33%) were working in professional sport, either with full-time professional athletes (21%), or elite funded athletes in institutes of sport (12%). Volunteers working in recreational sport made up 15% while 9% were semi-professional in part-time paid roles.

**Table 1:** (A) Employment and (B) education information presented for all respondents (total and group)

	Total	S&CC	A&P	SM&P	SP&B	SC
All respondents	241	114 (47)	42 (17)	41 (17)	24 (10)	20 (8)
A.						
Academic, university or school sport role	*66 (27)*	29 (12)	10 (4)	*11 (5)*	*10 (4)*	*6 (2)*
Professional: full-time paid position, full-time paid athletes	50 (21)	*37 (15)*	0 (0)	9 (4)	3 (1)	1 (0)
Volunteer, recreational club sport	35 (15)	4 (2)	*21 (9)*	6 (2)	2 (1)	2 (1)
Elite professional: full-time paid position, funded, amateur athletes (Institute)	30 (12)	15 (6)	1 (0)	4 (2)	7 (3)	3 (1)
Elite non-professional, part-time, regional or national athletes	30 (12)	16 (7)	5 (2)	7 (3)	0 (0)	2 (1)
Semi-professional: paid part-time position	22 (9)	9 (4)	2 (1)	3 (1)	2 (1)	*6 (2)*
Other	8 (3)	4 (2)	3 (1)	1 (0)	0 (0)	0 (0)
B.						
MSc/Masters	*96 (40)*	*51 (21*)	7 (3)	*20 (8)*	*13 (5)*	5 (2)
Degree/Hons	84 (35)	41 (17)	*17 (7)*	9 (4)	7 (3)	*10 (4)*
PhD	28 (12)	10 (4)	2 (1)	10 (4)	4 (2)	2 (1)
Diploma	27 (11)	9 (4)	13 (5)	2 (1)	0 (0)	3 (1)
Other	6 (2)	3 (1)	3 (1)	0 (0)	0 (0)	0 (0)

Data presented as number and percentage (*n* (%)) of all respondents. Italics represent the highest response for the column

*S&CC* strength and conditioning coaches, *A&P* athletes and players, *SM&P* sports medicine practitioners and physiotherapists, *SP&B* sports physiologists and biomechanists, *SC* sports coaches

Responses to all questions were analysed for all respondents (*n* = 241) and for each of the five demographic groups. There were no differences between group responses and total cohort, so data are presented and discussed for the total cohort.

The majority (87%) were qualified to degree level or higher, 40% had masters or MSc degrees and 12% had doctoral degrees. Most respondents (73%) reported to have a discipline specific professional qualification. Respondents reported to have been working in their specific discipline for an average of 8 years (range 0–36 years).

### Anatomy and name of the core

In response to the question on the anatomical region that comprised the core, half of the respondents (50%) identified the region between and including the pelvic and shoulder girdles and associated muscles and nerves (Table 2). Approximately, a quarter of respondents (27%) identified the region between the diaphragm and pelvic floor and associated muscles and nerves as the core, while for 18%, this was the lumbar spine, pelvis, hip joints and related muscles and nerves. Interestingly, more participants (45%) felt that the region should be called the trunk while 35% supported the term core and 18% preferred torso.

**Table 2:** Responses to the question of what (A) anatomic region makes up the core and (B) which term best describes this anatomical region

	Total
A.	
The spine and the associated muscles and nerves	5 (2)
The lumbar spine, pelvic and hip joints and associated muscles and nerves	43 (18)
The region between and including the pelvic and shoulder girdles and associated muscles and nerves	*120 (50)*
The region between and diaphragm and pelvic floor and associated muscles and nerves	65 (27)
Other	8 (3)
B.	
Torso	43 (18)
Trunk	*108 (45)*
Core	85 (35)
Upper limb	0 (0)
Other	5 (2)

Data presented as number and percentage (*n* (%)) of all respondents. Italics represent the highest response

### Methods of measuring core function

Respondents were asked to identify the most effective method of measuring core stability in a healthy, uninjured person. Almost a quarter (22%) reported that there was no effective method to test core stability. A number (43%) of the respondents proposed subjective assessment of core stability through observation. Of these, 17% suggested observation of sport-specific movement or exercise technique and 26%, observation of ground-based loaded barbell exercises. Objective assessments were proposed by 32% and included the timed isometric plank (19%), functional movement screen (9%) and isometric trunk bracing with biofeedback (4%).

### Core function and core stability training

#### Core stability and core strength (Fig. 1)

The majority believed that core strength is required for stability (mean 1.9, 95% CI 1.8–2.0, *p* < 0.001) and far fewer agreed that these were separate attributes (mean 2.6, 95% CI 2.4–2.7, *p* < 0.001) (Fig. 1). Most participants disagreed with the statement that core strength was required for athletic performance, but not everyday life (mean 3.9, 95% CI 3.7–4.0, *p* < 0.001).

#### The effectiveness of certain exercise categories on CST (Fig. 2)

The exercise categories deemed most effective in developing core stability for dynamic athletic performance were (Fig. 2) squats and Olympic lifts (mean 1.7, 95% CI, 1.6–1.8, *p* < 0.001) and farmers walk (mean 1.7, 95% CI 1.6–1.9, *p* < 0.001). Conversely, support was moderate to low for traditional core stability exercises, namely suspended compound exercises (mean 2.2, 95% CI 2.1–2.3, *p* < 0.001), isometric plank (mean 2.5, 95% CI, 2.4–2.6, *p* < 0.001), hanging leg raise (mean 2.8, 95% CI 2.7–2.9, *p* < 0.001) and instability abdominal exercises (mean 2.8, 95% CI 2.7–3.0, *p* < 0.001). Participants identified two exercise categories that were more ineffective than effective; abdominal bracing (mean 3.2, 95% CI, 3.0–3.3, *p* < 0.001) and sit-ups (mean 3.7, 95% CI, 3.5–3.8, *p* < 0.001).

#### The exercise selection criteria for effective CST (Fig. 3)

Correct movement pattern (mean 1.8, 95% CI 1.7–1.9, *p* < 0.001) was identified as most important exercise selection criteria for development of core stability for dynamic athletic performance (Fig. 3). Exercises characterised by forces that were equal to or greater than the force in the sport or event, were supported by 60% of the cohort (mean 2.4, 95% CI 2.3–2.5, *p* < 0.05). Most were either undecided or disagreed on the importance of velocity of movement (mean 2.6, 95% CI 2.5–2.8, *p* < 0.05) and sustained isometric contraction (mean 2.7, 95% CI 2.6–2.8, *p* < 0.05) in core stability exercises for athletic performance.

#### Ground-based free barbell exercises and trunk muscle activation (Fig. 4)

Most participants agreed that increases in external load in standing barbell exercises would increase trunk muscle activation (mean 2.0, 95% CI 1.9–2.1, *p* < 0.001) (Fig. 4). Equally important in this form of resistance training was correct postural control (mean 2.0, 95% CI 1.9–2.2, *p* < 0.001). Slow controlled movement (mean 2.8, 95% CI 2.7–2.9, *p* < 0.001) and increases in velocity (mean 2.6, 95% CI 2.5–2. 8, *p* < 0.001) of strength training exercises were not seen as important in eliciting trunk muscle activation in ground-based free barbell exercises.

Finally, results for the general questions on the application of core stability exercises are presented on Table 3. Most participants (85%) felt that it was appropriate to include specific exercises to train core stability in healthy, uninjured individuals. Less than half (45%) felt that it was effective to exercise the core stabilisers in isolation, while a majority (65%) agreed that core stability is developed during normal progressive exercise training.

**Table 3:** Answer to a series of questions about the application of core stability

		Total
Do you think it is necessary to include specific exercises to train core stability in a healthy, uninjured athlete’s exercise programme?	Yes	*206 (85)*
	No	30 (12)
	Do not know	5 (1)
Do you think it is possible to isolate and train the core stabilization system?	Yes	*120 (50)*
	No	82 (34)
	Do not know	39 (16)
Do you think it is effective to isolate and train the core stabilization system?	Yes	89 (37)
	No	*108 (45)*
	Do not know	44 (18)
Do you think that the core stability is automatically developed during normal, progressive exercise training?	Yes	*157 (65)*
	No	67 (28)
	Do not know	17 (7)

Data presented as number and percentage (n (%)) of all respondents. Italics represent the highest response for each question

## Discussion

Core stability training for healthy and athletic populations has been scrutinised and challenged in recent years in scientific literature [6, 7, 10, 13, 41–43]. Descriptions of the core by anatomic structures are entirely dependent on the chosen definition of core function [1]. The original narrow definition presented in early research focussed on the spinal region between the diaphragm and pelvis [44]. This approach identified muscular and neural dysfunction associated with back pain. Hence, core function was isolated to this region and proposed training intervention isolated the involved muscles. This approach did not transfer to healthy individuals and athletes where core function is obviously at the centre of dynamic movement characterised by force and velocity through the length of the body [10]. Core stability described by Fletcher (2016), ‘…is the kinetic link transferring torques between the upper and lower extremities in sporting actions’ [45]. Consequently, constituent anatomy of the core is described in the literature to reflect, i.e. region between and including pelvic and shoulder girdles and associated skeleton, muscles and nerves [1, 8]. Our survey results suggest this shift has permeated applied sports setting; half of the respondents agreed with this definition of the core while a quarter identified with the original description, i.e. structures between diaphragm and pelvic floor including muscles and nerves.

Surveys have been used effectively to assess nutrition knowledge [46] and understanding of scientific training principles [47] in the workplace. Response rate to our survey (*n* = 241) was good in comparison to similar surveys which gathered information from both athletes (Wade et al., *n* = 57) [48] and people working in sport (Taylor et al., *n* = 28) [49], (Durell et al., *n* = 137) [47] and (Torres-McGehee et al., *n* = 579) [46]. Furthermore, the representative quality of our cohort is reflected by the spread of respondents, with 33% in full-time professional positions, either working with professional athletes (21%) or full-time Institute of sport athletes (12%). A quarter (27%) were involved in sport in an academic setting, either school or university and a quarter (27%) were in non-professional roles, either volunteering (15%) or part-time (12%). The majority were qualified to degree level (87%) and half had postgraduate degrees (52%). Most had an industry-specific qualification and on average were well experienced (mean 8 years) in their discipline. The cohort is therefore representative of people working and participating in sport. Furthermore, they were reasonably well informed, indicating survey results that represent unbiased perceptions of the wider population.

Our survey investigated perceptions around core stability and core strength (Fig. 1). The majority believed that core strength is required for stability and far fewer agreed that these were separate attributes. In a comprehensive review Hibbs et al. [1] concluded that these two terms had yet to be clearly defined, in fact they failed to identify any characteristics that differentiated exercises for core strength and core stability. These researchers reviewed studies that investigated core stability in response to loaded resistance exercises and traditional core stability exercises. A later systematic review proposed a five-level core exercise classification system that progressed from traditional core exercises to noncore free weight exercises [6]. Interestingly the fourth classification level was free weight exercises defined as ‘dynamic, externally loaded, intent to activate lower body and core muscles’. Both these reviews suggest that the concept of strength in the term core strength relates to the overarching nature of the exercise, rather than the impact on or adaptation in the core stabilization system.

While core strength and core stability may well be viewed by some in our survey as separate entities, this has yet to be demonstrated scientifically [1]. The selection of exercises used to develop core stability for healthy function can range from low load, minimal range of movement, abdominal bracing exercises to dynamic, loaded resistance exercises [6]. Research has not been able to identify and describe adaptations that occur in muscles responsible for stabilising the core as a consequence of different exercise modes [1, 12]. It is recognised though that effective core stability is the control of movement, including high force and high velocity movement, generated by interaction between axial and appendicular skeletons [5, 7, 8]. Most survey responses disagreed with the statement that core strength was required for athletic performance, but not everyday life. This demonstrated alignment with the principle that core stability underpins both healthy function and dynamic athletic performance. In effect core strength and core stability are synonyms and are used accordingly in the literature [1, 5, 23]. This is reflected in the survey question seeking to determine whether core stability and strength are separate attributes. Responses were mixed with just over half (57%) in agreement and the rest either undecided (16%) or in disagreement (27%).

In our survey questions that assessed support for exercise categories most effective in developing core stability for dynamic athletic performance, there was clearly more support for functional, loaded exercises (Fig. 2). Squats and Olympic lifts and farmers walk that engage the full kinetic chain. Conversely support was moderate to low for traditional, non-functional core stability exercises, namely suspended compound exercises, isometric plank, hanging leg raise, and instability abdominal exercises. Two exercise categories, namely abdominal bracing and sit-ups, were regarded as ineffective rather than effective, The survey results therefore reflect the many reviews that highlighted a lack of evidence to support traditional CST for healthy individuals and recommended loaded, dynamic exercises that engage the full kinetic chain [1, 6, 7, 12–14, 45].

Correct movement pattern was identified as most important exercise selection criteria for development of core stability for dynamic athletic performance (Fig. 3). Exercises characterised by forces that were equal to or greater than force in the sport or event, were supported by 60% of the cohort. Most were either undecided or disagreed on whether velocity of movement and sustained isometric contraction were important in core stability exercises for athletic performance. Kibler et al. (2006) accurately describes the exercise criteria for effective CST: ‘integrated activation of multiple segments’ providing ‘force generation’ that produces ‘interactive movement’ characterised by ‘proximal stability and distal mobility’ [5]. Core stability development is therefore integral to all dynamic exercise training and sports specific movement, while quality of training effect is determined by specificity of movement, forces and velocity.

There is growing evidence in the literature that external load in free barbell exercises performed in a standing position is related to muscle activation of trunk stabilisers [29, 30, 33, 34, 37, 50]. Impact of this stimulus on core stability in dynamic athletic performance is more difficult to demonstrate. In a recent systematic review, Prieske et al. (2016) reported a large effect for CST on trunk muscle strength measured by timed isometric plank, compared to no or only regular sports training [12]. When compared to alternative training, such as whole-body strength training, CST had a small sized effect on trunk muscle strength. CST had a small sized effect on muscle strength (e.g. Squat 1RM), a medium sized effect on muscle power (e.g. countermovement jump) and a small sized effect on athletic performance (e.g. 5000 m run time). They concluded that CST for healthy individuals, in the absence of any other fitness training, would increase trunk muscle strength. However, when combined with other training, such as whole-body strength training, CST is not effective. They also propose that increases in trunk muscle strength from CST, has limited effect on physical fitness and athlete performance in trained individuals. Findings from the survey indicate that this information has begun to inform applied practice (Fig. 4). Most agreed that increases in external load in standing barbell exercises would increase trunk muscle activation. Equally important in this form of resistance training was correct postural control.

The survey included a series of questions (yes/no/do not know) investigating perceptions on the application of CST for dynamic athletic performance (Table 3). Most (85%) of the cohort felt it necessary to include specific exercises to train core stability in healthy, uninjured athletes. With reference to traditional CST, two questions were asked; whether it was possible to isolate and train the core stabilization system, and whether this approach was effective. Half of the group believed that this was possible, 34% felt not and the rest were undecided (16%). The isolated training approach was regarded as not effective by 45%, and 37% were supportive. Prieske’s review highlighted growing evidence that specific, traditional CST is ineffective in healthy individual and athletes [12]. They also that reported that regular sports training and commonly used supplementary training, such as whole-body strength training, presents superior stimuli, that adhere to the overload training principle [28], for development of core stability in this population. Most survey respondents (65%) concurred with this by agreeing that core stability is developed through normal, progressive exercise training. The perception in applied practice conflicts with scientific literature with regards effectiveness of traditional core stability exercises for athletic performance. The majority (85%) of survey respondents believed that specific exercises were required to train core stability and half supported the use of exercises that isolated trunk stabilisers.

A limitation of the survey was the method of recruiting participants through email and direct messaging on an online professional community platform (LinkedIn). Emails and notifications may have been filtered to spam or junk folders and not reached intended participants. Participants were directed to an online survey, which may have served as a deterrent. Despite this, the number and quality of participants was good in comparison to similar surveys. A further limitation may well have been the inconsistency of prevailing terminology around the topic of CST and broader area of exercise and fitness. Steps were taken to adhere to the most commonly used terms from the scientific literature in the survey.

## Conclusion

The survey has provided evidence that a revised, more functional definition of core function and constituent anatomy described in the literature is starting to be used in the practical setting. Almost half (45%) of the respondents preferred trunk as the name for this anatomical region over core (35%). The absence of a valid objective method of measuring core function (22%) means that the most effective way is through observation (43%) of exercise and athletic movement. A quarter (26%) proposed subjective assessment of movement in upright loaded resistance exercises as the most effective method of measuring core function. This coincides with the strong shift in perceptions towards more functional approach to core stability training for dynamic athletic performance. Loaded exercises in an upright position, such as barbell squat and farmers walk, were viewed as effective training methods as proposed in the literature [7, 8, 14]. Core stability as an integrated, functional motor task [7], with training reflecting this according to movement patterns [14], forces [7], torque and velocity [8], appear to be guiding practice in the workplace according to the survey. These findings along with strong support for developing core stability through normal progressive exercise training, means we found in favour of our hypothesis. Some support remained for traditional CST through specific exercises (85%) and the isolation approach (50%). Our findings lead to the following recommendations: Research to continue into efficacy of activating trunk stabilisers through selected sport specific and supplementary training modalities, including compound, loaded strength exercises. Continue to investigate the transfer of training induced trunk muscle activation to functional performance, specifically functional stability.

## Additional file

Additional file 1: Core Stability Survey. (PDF 103 kb)

## Abbreviations

1RM: 1 Repetition maximum
CST: Core stability training
MSc: Master of Science
S&CC: Strength and conditioning coaches
A&P: Athletes and players
SM&P: Sport medicine practitioners and physiotherapists
SP&B: Sport physiologists and Biomechanists
SC: Sport coaches
