# Supplementary material for: Contemporary perspectives of core stability training for dynamic athletic performance: a survey of athletes, coaches, sports science and sports medicine practitioners
Source: Sports Med Open. 2018 Jul 16;4:32. doi: 10.1186/s40798-018-0150-3 (PMC6047949; doi:10.1186/s40798-018-0150-3)
Supplement: Supplementary file 1 — Core Stability Survey. (PDF 103 kb) [file 40798_2018_150_MOESM1_ESM.pdf]

Core Stability (copy)

Page 1: Page 1

There have been a number of recent reviews highlighting the lack of consensus around the topic of core stability. There is however agreement that core stability is important in everyday life and dynamic sporting activity. The confusion lies in the most effective manner of developing and measuring core stability.

This motivated me to embark on a PhD a few years ago looking at neuromusclar funtion of the trunk in the loaded squat in the hope of shedding some light on this approach for developing core stability.

An obvious related question is: How do people working in sport view core stability and its development for dynamic athletic performance?

I appreciate your time in completing this short survey (20 min) which will focus on ***core stability for dynamic athletic performance***.

Page 2

Section 1: Demographics

1 What is your MAIN discipline? *Optional*

- Sports Medicine Practitioner
- Sports Physiotherapist
- Masseur / Soft Tissue Therapist
- Strength and Conditioning Coach
- Sports Physiologist
- Sports Psychologist
- Performance Nutritionist
- Biomechanist
- Performance Analyst
- Sports Coach
- Athlete / Player
- Other

2 What level of sports performance are you involved in? *Optional*

- Professional, full-time paid position working with full-time paid atheletes
- Semi-professional, paid position but not enough to make a living
- Elite professional, full-time paid position working with funded and amateur athletes (Institute)
- Elite non-professional, part-time working with regional or national selected athletes
- Volunteer in recreational club sport
- Academic, university or school sport role
- Other

3 Please indicate below which describes most accurately where you do most of your work. *Optional*

- Team sport
- Individual athletes
- Combination of team and individual athletes

4 What is your highest academic qualification? *Optional*

- PhD
- MSc or Masters
- Degree or Honours degree
- Diploma
- Other

4.a If you selected Other, please specify:

4.b Do you have a professional qualification linked to your discipline? *Optional*

- Yes
- No

4.c How many years have you been working in your current discipline?

Section 2: Core Stability

5 Which statement below **do you think** describes the core most accurately?
Optional

- ○ Passive spinal column, active spinal muscles and neural control unit
- ○ Lumbo-pelvic-hip complex
- ○ Axial skeleton between the pelvic and shoulder girdle including rib cage, spinal column and associated musculature
- ○ Local stabilizers control intersegmental spinal movement while global stabilizers develop intra-abdominal pressure
- ○ Other

5.a If you selected Other, please specify:

6 Do you think it is necessary to include specific exercises to train core stability in a healthy, uninjured ***non athlete's*** exercise programme?

- ○ Yes
- ○ No
- ○ Don't know

7 Do you think it is necessary to include specific exercises to train core stability in a healthy, uninjured ***athlete's*** exercise programme?

- Yes
- No
- Don't know

8 Do you think that the development of core stability can ***prevent back pain***?

- Yes
- No
- Don't know

9 Do you think that certain ***lower limb overuse injurie***s are caused by ***malfunctioning*** core stabilization system?

- Yes
- No
- Don't know

10 Do you think it is ***possible*** to isolate and train the core stabilization system?

- Yes
- No
- Don't know

11 Do you think it is ***effective*** to isolate and train the core stabilization system?

- Yes
- No
- Don't know

12 Do you think that the core stabilization system is ***automaticall***y developed during normal, ***progressive*** exercise training?

- Yes
- No
- Don't know

13 Do you believe that trunk muscle activation measured by ***surface electromyography*** is ***reflective of performance*** of the core stabilization system?

- Yes
- No
- Don't know

14 Please rate the following ***categories of exercise*** on thier ***effectiveness*** in developing core stability ***for dynamic athletic performanc***e? *Optional*

Please don't select more than 1 answer(s) per row.

Please select at least 8 answer(s).

	1 Least effective	2	3	4	5 Most effective
Isolated abdominal bracing	☐	☐	☐	☐	☐
Isometric held exercises such the plank	☐	☐	☐	☐	☐
Dynamic abdominal exercises such as sit-ups	☐	☐	☐	☐	☐
Dynamic inverted exercises such as hanging leg raise	☐	☐	☐	☐	☐
Suspended compound exercises using systems such as the TRX	☐	☐	☐	☐	☐
Instability abdominal exercises performed on Swiss ball	☐	☐	☐	☐	☐
Functional exercises such as farmers walk	☐	☐	☐	☐	☐
Loaded free barbell exercises such as Squats and Olympic lifts	☐	☐	☐	☐	☐

15 Please rate how strongly you agree or disagree with the following statements as they relate to determining ***exercise selection*** for the

development of core stability for ***dynamic athletic performanc***e. *Optional*

Please don't select more than 1 answer(s) per row.

Please select at least 4 answer(s).

	1 Strongly agree	2 Agree	3 Neither agree nor disagree	4 Disagree	5 Strongly disagree
The exercise must subject the athlete to forces equal to or greater than expected in the sport or event	☐	☐	☐	☐	☐
The exercise must emphasize correct movement pattern above all else	☐	☐	☐	☐	☐
The exercise must subject the athlete to velocity of movement equal to or greater than expected in the sport or event.	☐	☐	☐	☐	☐
The exercise must develop capacity for sustained isometric contraction	☐	☐	☐	☐	☐

16 What term do you believe best describes the anatomical region that this

survey has been dealing with? *Optional*

- Core
- Trunk
- Torso
- Upper limb
- Other

16.a If you selected Other, please specify:

17 Please rate how strongly you agree or diagree with the following statements as they relate specifically to ***ground based loaded free barbell exercises*** (Squats and Olympic weightlifting exercises)***.***

Please don't select more than 1 answer(s) per row.

Please select at least 4 answer(s).

	1 Strongly agree	2 Agree	3 Neither agree nor disagree	4 Disagree	5 Strongly disagree
Trunk muscle activation will increase with increases in velocity of movement.	☐	☐	☐	☐	☐
Trunk muscle activation is dependent on correct postural control	☐	☐	☐	☐	☐

Trunk muscle activation is enhanced by slow controlled movement	☐	☐	☐	☐	☐
Trunk muscle activation will increase with increases in external load	☐	☐	☐	☐	☐

18 What is the most effect method of measuring core stability in a healthy, un-injured person?

19 Please rate how strongly you agree or disagree with the following statements.

Please don't select more than 1 answer(s) per row.

	1 Strongly agree	2 Agree	3 Neither agree ror disagree	4 Disagree	5 Strongly disagree
Core strength is required for core stability	☐	☐	☐	☐	☐
Core strength and core stability are separate attributes	☐	☐	☐	☐	☐

Core strength is required for dynamic athletic performance but not everyday life	☐	☐	☐	☐	☐
Core stability is dependent on neural timing and muscular coordination rather than core strength	☐	☐	☐	☐	☐

Page 3

Thank you for completing the survey.
